# METTL14 Facilitates the Metastasis of Pancreatic Carcinoma by Stabilizing LINC00941 in an m6A-IGF2BP2-Dependent Manner

**DOI:** 10.7150/jca.84070

**Published:** 2023-04-17

**Authors:** Jiawei Lu, Lanting Yu, Ni Xie, Ying Wu, Baiwen Li

**Affiliations:** Department of Gastroenterology, Shanghai Key Laboratory of Pancreatic Diseases, Shanghai General Hospital, Shanghai Jiao Tong University School of Medicine, Shanghai 201620, China.

**Keywords:** pancreatic carcinoma, METTL14, metastasis, LINC00941, IGF2BP2, N6-methyladenosine

## Abstract

Pancreatic adenocarcinoma (PC), one of the most fatal diseases, usually generates a poor prognosis in advanced stages. N6-methyladenosine modification has emerged as a crucial participant in tumor development and recurrence. Methyltransferase-like 14 (METTL14), as a core member of methyltransferases, is involved in tumor progression and metastasis. However, the potential mechanism by which METTL14 regulates long noncoding RNAs (lncRNAs) in PC remains unclear. RNA immunoprecipitation (RIP), methylated RNA immunoprecipitation quantitative PCR (MeRIP-qPCR), and fluorescence in situ hybridization (FISH) were used to explore the underlying mechanisms. In our study, we found that METTL14 expression was upregulated in PC patients, and was associated with poor prognosis. In vitro and in vivo experiments, knocking down METTL14 suppressed tumor metastasis. RNA-seq and bioinformatics analyses were used to identify LINC00941 as the downstream target of METTL14. Mechanistically, LINC00941 was upregulated by METTL14 in an m6A-dependent way. LINC00941 was recruited and recognized by IGF2BP2. METTL14 enhanced the affinity of IGF2BP2 for LINC00941, while IGF2BP2 promoted the stabilization of LINC00941, which contributed to the migration and invasion of PC cells. Overall, our research revealed that METTL14 promoted the metastasis of PC through m6A modification of LINC00941. Targeting the METTL14-LINC00941-IGF2BP2 axis may provide promising therapeutic approaches for PC.

## Introduction

Pancreatic carcinoma (PC), the most lethal malignant neoplasm of the alimentary system, is difficult to diagnosis early, has a poor probability of successful surgical resection, and continues to be a serious health problem worldwide 1. Pancreatic adenocarcinoma is the third leading cause of death worldwide, but it has the worst five-year survival rate among all cancers 2. Over the past few decades, despite significant advances in pancreatic cancer treatment, including curative surgery, radiotherapy and chemotherapy, immunotherapy and targeted therapy, pancreatic carcinoma outcomes remain unfavorable 3. Therefore, there is a pressing need to elucidate the underlying cellular and molecular processes of PC for diagnostic and therapeutic purposes.

Epigenetics is the study of the heritable modification of gene expression or cellular phenotype that does not affect the DNA sequence. RNA methylation modifications are especially crucial among the several epigenetic alterations such as histone acetylation, and DNA methylation 4. N6-adenosine methylation (m6A), N1-adenosine methylation (m1A), and cytosine hydroxylation (m5C) and are several common internal modifications of mRNA, among which m6A is the most abundant and significant posttranscriptional modification in eukaryotes 5. m6A modification is the process by which methyl transferase attaches a methyl group to the N6 position of adenosine in RNA. Many studies have indicated that m6A has significant impacts on the translation, stability, and localization of RNA and the splicing of pre-mRNA 6, 7. The process of m6A modification is reversible and dynamic, with methyltransferase complexes (writers) installing m6A on RNA and demethylases (erasers) removing it. The methyltransferase complexes are methyltransferase-like 14 (METTL14), methyltransferase-like 3 (METTL3), vir-like m6A methyltransferase-associated (KIAA1429), Wilms tumor 1 associated protein (WTAP), and methyltransferase-like 16 (METTL16), whereas fat mass and obesity-related protein (FTO) and alkylation repair homolog protein (ALKBH) are demethylases 5. In addition, there are a number of m6A regulators called reader proteins including insulin-like growth factor 2 mRNA binding protein (IGF2BP) and YTH domain-containing family protein (YTHDF) whose functions are to identify bases undergoing m6A modification to regulate the downstream expression of RNA. Accumulating proofs indicated that m6A was involved in numerous biological functions such as stem-cell renewal and differentiation, circadian rhythm regulation 8, the innate immune response, and other homeostatic mechanisms 9. Therefore, diseases such as cancer might arise when m6A is dysregulated. For example, ALKBH5 suppresses the progression of colorectal cancer by reducing the methylation of PHF20 mRNA 10. As an m6A demethylase, FTO plays a crucial role in enhancing chemoresistance in colorectal cancer cells and research has revealed that inhibiting FTO might restore 5-FU sensitivity in chemoresistant CRC cells 11. Moreover, previous studies 12-14 demonstrated that tumor progression was correlated with lncRNA stability regulated by m6A modification.

Long noncoding RNAs (lncRNAs) refers to a kind of functional noncoding RNA transcripts with more than 200 nucleotides 15. Increasing evidence demonstrates that lncRNAs are involved in different physiopathological processes during the onset of neoplasia 16, 17. Functionally, lncRNAs can coordinate the pathogenesis of human cancers and the proliferation and differentiation of human cancer cells 18. LINC00941, which has just recently begun to be investigated, has emerged as an oncogene and been found to contribute to the development of tumorigenesis and clinicopathological characteristics of cancers 19-21. LncRNA have activating effects on several tumorigenic signaling pathways. For instance, LINC00941 has been indicated to accelerate glycolysis in pancreatic cancer by activating the Hippo pathway 22. However, the connection between LINC00941 and m6A regulators has never been explored.

In this study, we clarified the effects of METTL14 on the metastasis of pancreatic cancer. METTL14 significantly promoted the migration and invasion of PC cells in vitro and in vivo. Next, we identified LINC00941, a potential noncoding RNA, as its downstream target and proved its tumor-promoting functions. Mechanistically, we demonstrated that METTL14 elevated the m6A level of LINC00941, which recruited IGF2BP2 to recognize and enhance the stability of LINC00941. As a result, this work is anticipated to confirm the molecular mechanism of METTL14 in PC and provide an updated theoretical understanding for the improvement of PC treatment.

## Materials and methods

### Immunohistochemistry (IHC) and in situ hybridization (ISH)

Tissue microarrays (TMAs) were obtained from 42 PC patients, who underwent therapeutic resection for pancreatic cancer in Shanghai General Hospital. And the postoperative pathological diagnosis was pancreatic cancer. Patients lacking clinical information were excluded. The use of clinical samples was authorized by the Ethics Committee of Shanghai General Hospital. Detailed clinic parameters of enrolled patients were listed in Table S9. The TMA cohort was employed to establish the relationship between METTL14/LINC00941 and PC patient prognosis, while paraffin-embedded tissues were used to validate the association between METTL14 and LINC00941. The IHC and ISH score was calculated by adding the staining intensity score and positive staining rate grade. The score was independently assessed by two proficient pathologists. The intensity scores of 0, 1, 2, and 3 were indicative of negative, weak positive, moderate positive and strong positive staining, respectively. Similarly, positive rate grades of 0, 1, 2, 3, and 4 indicated regions of positive staining ranging from 0 to 10 %, 11 to 25 %, 26 to 50 %, 51 to 75 %, and 76 to 100 % of the sample, respectively. A final score of five or more is considered high expression, while a score of four or less is considered low expression.

### Cell lines and cell culture

Human PC cell lines were obtained from the Type Culture Collections of the Chinese Academy of Science (Shanghai, China). PANC-1 and MIAPaCa-2 cells were cultured in DMEM medium (Gibco, USA), while SW1990, AsPC-1, BxPC-3, and HPDE6-c7 cells were cultured in RPMI-1640 medium (Gibco, USA). Both media were supplemented with 10 % fetal bovine serum (FBS), 100 U/ml penicillin and 100 μg/ml streptomycin. Cells were grown at 37 °C in a cell culture incubator containing 5 % CO2.

### Plasmid and lentivirus production

Short hairpin RNAs (shRNAs), small interfering RNAs (siRNAs), and overexpression vectors were generated by RiboBio (Guangzhou, China). Transfection of siRNA, shRNA and plasmid was performed with LipofectamineTM 3000 (Invitrogen, USA). The effectiveness of transfection was assessed using western blot and RT-qPCR analysis. All the target sequences are displayed in Table S1.

### RNA extraction and real-time PCR analysis

Total RNA was extracted with RNAiso Plus (Takara, Japan) and reverse transcribed using a HyperScript III RT SuperMix for qPCR with gDNA Remover Kit (R202, EnzyArtisan, China). cDNA was mixed with Universal SYBR qPCR Mix (Q204, EnzyArtisan, China) along with specific primers for RT-qPCR. All operations were carried out according to the product instructions. Glyceraldehyde 3-phosphate dehydrogenase (GAPDH) was utilized as the internal reference, and the relative expression in the control group was determined by the 2^-ΔΔCt^ method. The primers involved in our research are listed in Table S2.

### Western blotting

Harvested cells were digested in RIPA lysis buffer (Beyotime, Shanghai, China), and then proteins in the whole-cell lysates were separated and electroblotted onto PVDF membranes (Millipore, America). Membranes were blocked for 1 hour with 5 % nonfat milk in TBST before incubating overnight at 4 °C with specific primary antibodies. Immunoblot bands were detected with a detection reagent (Tanon, shanghai, China). The detailed antibody information is listed in Table S3.

### Wound healing assay

PC cells were seeded in 6-well plates. When the cells were 80 %-90 % confluent, a 200 μL micropipette tip was used to create a scratch wound in the center of each well. Images of the assay were collected at 0 h and 36 h post-injury. The width of the healing wound was measured and compared to the initial width.

### Transwell assays

PC cells (5×10^4^) were seeded in the top chamber for Transwell migration experiments. The membrane in the top chamber was covered with Matrigel (BD Biosciences, USA) before the Transwell invasion assay. After 24 h or 48 h of incubation, cells that did not pass through the pores in the membrane were removed by wiping with cotton. Cells on the bottom of the chamber membrane were fixed with 4 % paraformaldehyde and stained with 0.5 % crystal violet. Subsequently, four random fields per chamber were selected for imaging.

### Animal experiments

All experimental procedures in this study were authorized by the Animal Care Committee of Shanghai General Hospital. PANC-1 cells were stably transfected with LV-METTL14, LV-NC, sh-METTL14, sh-NC, or sh-LINC00941. A mixture of 2×10^6^ cells and 100 μL PBS was injected into the spleen of every BALB/c nude mouse. After two months of housing in a sterile environment, the mice were sacrificed. Their liver tissues were removed for hematoxylin and eosin (HE) staining.

### RNA sequencing and bioinformatics analysis

RNA sequencing was performed on shMETTL14 and control PANC-1 cells using HiSeq-2000 (Qiantang Biotechnology, Suzhou). The criteria for differential gene expression were *p* < 0.05 and |Log_2_Fold change (FC)| > 1. Analysis of signaling pathways was subsequently performed using KEGG databases. RNA sequencing data from PC patients were downloaded from The Cancer Genome Atlas (TCGA) database and the Genotype-Tissue Expression Project (GTEx). Pearson correlation analysis (|coefficients| > 0.3, and *p* < 0.001) was conducted to identify m6A-related lncRNAs. Univariate Cox regression analysis with a threshold of *p* < 0.05 was used to identify m6A-related lncRNAs with prognostic value.

### Immunofluorescence (IF) and Fluorescence in situ hybridization (FISH)

PANC-1 and AsPC-1 cells were grown on confocal dishes overnight. Then the cells were incubated with METTL14 or IGF2BP2 anti-body at 4 °C overnight. The next day, cells were fixed with paraformaldehyde and hybridized with the LINC00941 probe, which was used to detect the lncRNA. Signals were observed by confocal microscopy (Leica, Germany).

### RNA immunoprecipitation (RIP)

RIP assays were conducted with an RNA Immunoprecipitation Kit (Geneseed, Guangzhou, China), according to the manufacturer's instructions. The protein A + G beads were first incubated with antibodies. Then, the cells were lysed with lysis buffer of the kit and incubated overnight with protein A + G beads bound to antibodies. Finally, the RNA was purified, and RT-qPCR analysis was performed.

### MeRIP-qPCR

MeRIP-qPCR was performed according to the manufacturer's instructions (C11051-1, RiboBio, China). Briefly, 100 μg of total RNA was sheared into approximately 200-nt fragments. One tenth of the fragmented RNA was preserved as the input control for future experiments. Magnetic beads A/G were incubated with 5 μg of an anti-m6A antibody or rabbit IgG for 0.5 h at room temperature with rotation. The remaining fragmented RNA was incubated with antibody-conjugated beads for two hours at 4 °C. After three washes and two elutions, the methylated RNA was purified by a Magen HiPure Serum/Plasma miRNA Kit (R4317-03, Magen, China) and reverse transcribed to cDNA. The results were analyzed by RT-qPCR.

### Treatment with methylation inhibitors

PC cells were treated with 3-deazaadenosine (DAA, MedChemExpress, USA) at a concentration of 100 μmol/L for 24 h, and then the expression of LINC00941 was analyzed by RT-qPCR.

### RNA stability assays

PC cells were seeded in 12-well plates for 12 hours, and then dosed with actinomycin D (5 μg/mL, HY-17559, MedChemExpress, USA) at 0, 3, and 6 hours and before collection. RT-qPCR was utilized to identify RNA level variations.

### Statistical analysis

All data were statistically analyzed with GraphPad Prism 9.0 and SPSS 20.0 software. The significance of differences between two groups were analyzed using a two-tailed t test. The chi-square test was used to determine the correlations between clinicopathological values and tissue staining scores. Survival curves were generated using the Kaplan-Meier method and compared using the log-rank test. *p* < 0.05 was considered statistically significant.

## Results

### High METTL14 expression is correlated with poor prognosis in PC patients

**Figure 1:**
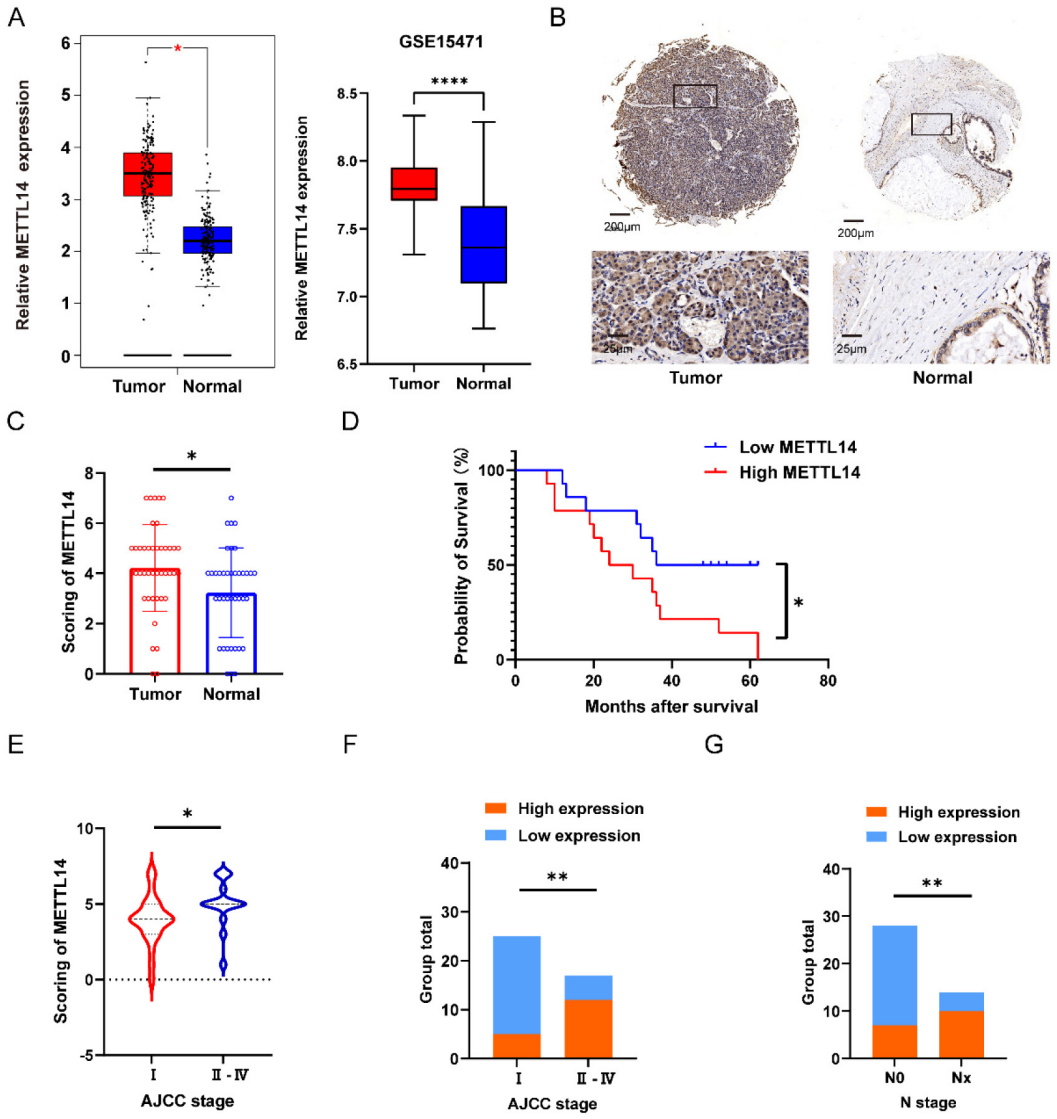
METTL14 was upregulated in PC and associated with poor prognostics in PC patients. (A) Expression level of METTL14 between tumor and normal group in TCGA-GTEx and GSE15471. (B) IHC staining results of METTL14 expression in PC and para-carcinoma tissue (Top image, scale bar, 200 μm; magnification, 50×; bottom image, scale bar, 50 μm; magnification, 400×). (C) IHC staining scoring of 42 pairs of tumor and adjacent tissues in PC patients. (D) The survival of PC patients was associated with the expression of METTL14 in TMA. (E-F) AJCC stage was associated with the expression of METTL14 in PC patients. (G) The lymph node metastasis was associated with level of METTL14 in PC patients. * *p* < 0.05, ** *p* < 0.01, *** *p* < 0.001, **** *p* < 0.0001.

To investigate the potential role of METTL14 in pancreatic cancer, 42 pairs of tumor and adjacent tissues from PC patients were evaluated by IHC staining. The staining results suggested that METTL14 was expressed at a higher level in PC tissues than in normal tissues (Figure 1B). The results were consistent with the analysis of METTL14 expression in the TCGA and GTEx database as well as the GSE15471 dataset (Figure 1A). Next, we examined the expression of METTL14 in PC cell lines, and the findings indicated that PC cells expressed higher levels of METTL14 than normal pancreatic epithelial cells (HPDE6-c7) (Figure S1A-B). To investigate the clinicopathological features of METTL14 in pancreatic cancer, we scored the staining in the TMA (Figure 1C). PC patients with higher METTL14 expression had shorter overall survival times (Figure 1D). Further study revealed a favorable correlation between the METTL14 levels and N stage (*p* < 0.01) and American Joint Committee on Cancer (AJCC) stage (*p* < 0.01) (Figure 1E-G), suggesting that METTL14 might regulate the metastasis of pancreatic cancer. Moreover, tumor location and ages of PC patients showed significant relevance to the expression of METTL14 (Table 1), which indicated METTL14 was closely related with tumor progression and could be utilized as a prognostic marker in pancreatic cancer.

### METTL14 promotes the migration of PC cells in vitro

**Figure 2:**
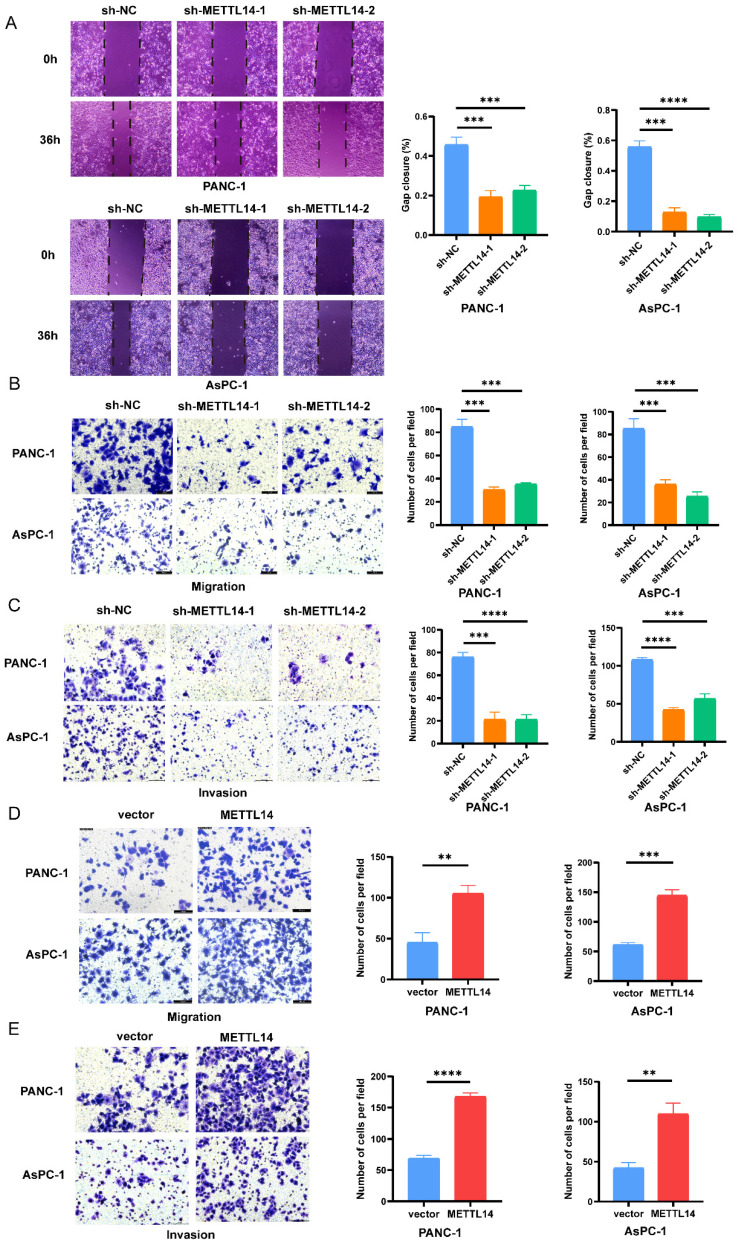
METTL14 promoted the migration and invasion in PC cells in vitro. (A) Knockout METTL14 inhibited scratch healing in PC cells. (B-C) The migration and invasion abilities of PANC-1 and AsPC-1 were reduced after knockdown of METTL14 in transwell assays (200×). (D-E) METTL14 enhanced the migration and invasion abilities of PANC-1 and AsPC-1 in transwell assays (200×). * *p* < 0.05, ** *p* < 0.01, *** *p* < 0.001, **** *p* < 0.0001.

We next tried to explain the mechanism by which METTL14 promotes the malignant phenotype of PC cells. First, PANC-1 and AsPC-1 cell lines with stable knockout and overexpression of METTL14 were established. The RT-qPCR results confirmed that the METTL14 level was considerably decreased in the PC cells with stable knockout and increased in those with stable overexpression (Figure S2A-B, D-E). Moreover, knockdown and overexpression of METTL14 were validated also by western blot (Figure S2C, F). Then, the results of a wound healing assay showed that knockdown of METTL14 significantly suppressed the migration of PC cells (Figure 2A). In contrast, the wound coverage was accelerated in the wells containing cells with stable METTL14 overexpression than in those containing control cells (Figure S3A-B). The same trend was observed in the Transwell assays. Moreover, the migration and invasion abilities of PC cells were reduced after METTL14 knockdown (Figure 2B-C). In METTL14-overexpressing cells, as expected, an increase in the migration ability as well as a marked increase in invasion were observed compared with those in the corresponding control cells (Figure 2D-E). The above results showed the oncogenic role of METTL14, especially in promoting tumor invasion and the migration of PC cells.

### METTL14 promotes the metastasis of PC cells in vivo

**Figure 3:**
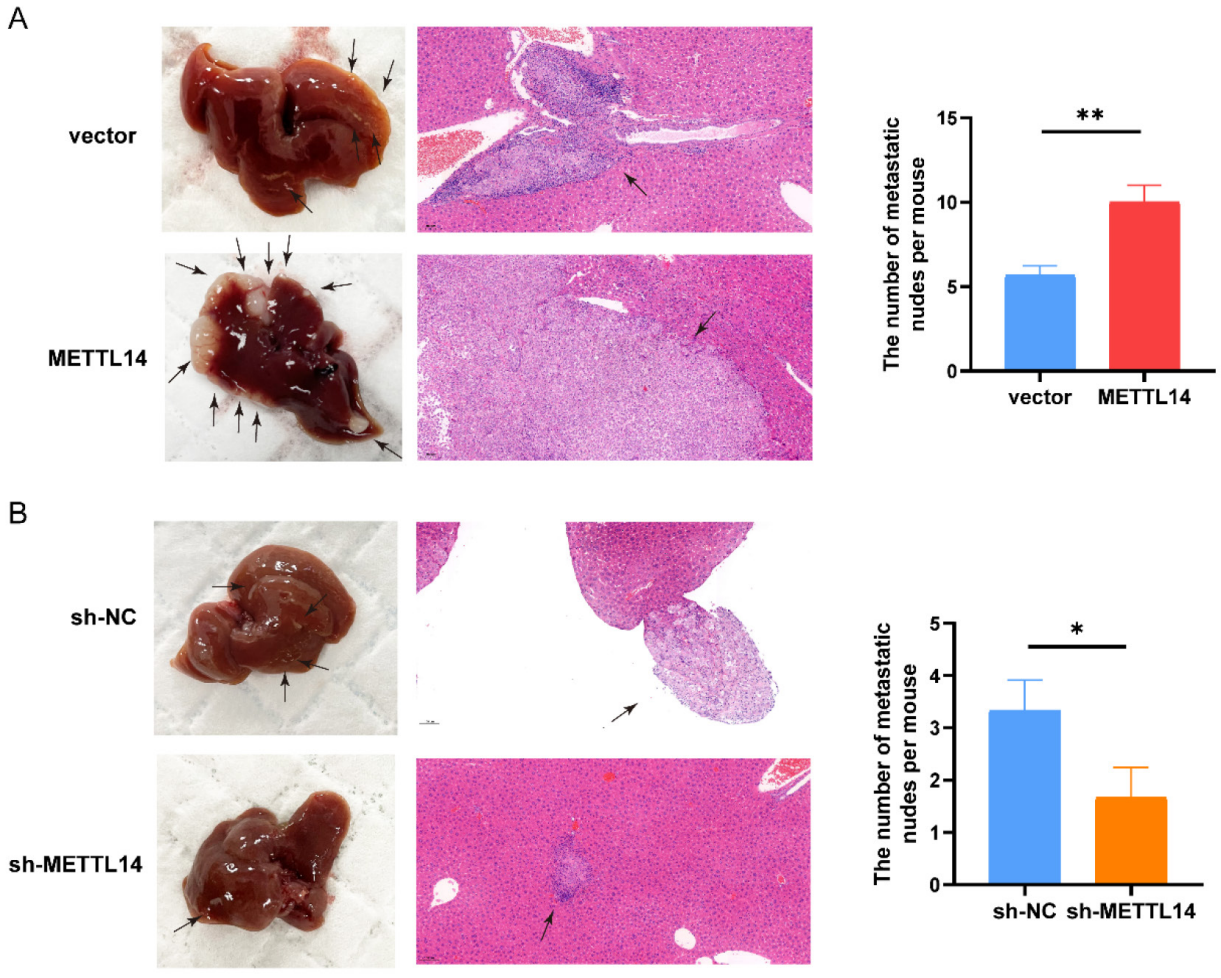
METTL14 promoted the metastasis of PC cells in vivo. (A) Representative images and HE staining of metastatic sites in the liver of nude mice injected with vector or METTL14 lentiviruses cells. (B) Representative images and HE staining of metastatic sites in the liver of nude mice injected with sh-NC or sh-METTL14 cells. * *p* < 0.05, ** *p* < 0.01.

Next, we investigated the functions of METTL14 in vivo by establishing a liver metastasis model. We divided nude mice randomly into four groups. Then, we generated OE-METTL14 and NC PANC-1 cells and injected them into the spleens of the nude mice. Two months after cell injection, the mice were sacrificed. Compared to the control mice, mice injected with METTL14-overexpressing cells exhibited more liver metastasis (Figure 3A). In addition, mice injected with METTL14 knockdown cells developed fewer liver metastatic nodules than those injected with control PANC-1 cells (Figure 3B). The results further highlighted the important function of METTL14 in PC metastasis in vivo.

### LINC00941 is the downstream target methylated by METTL14

**Figure 4:**
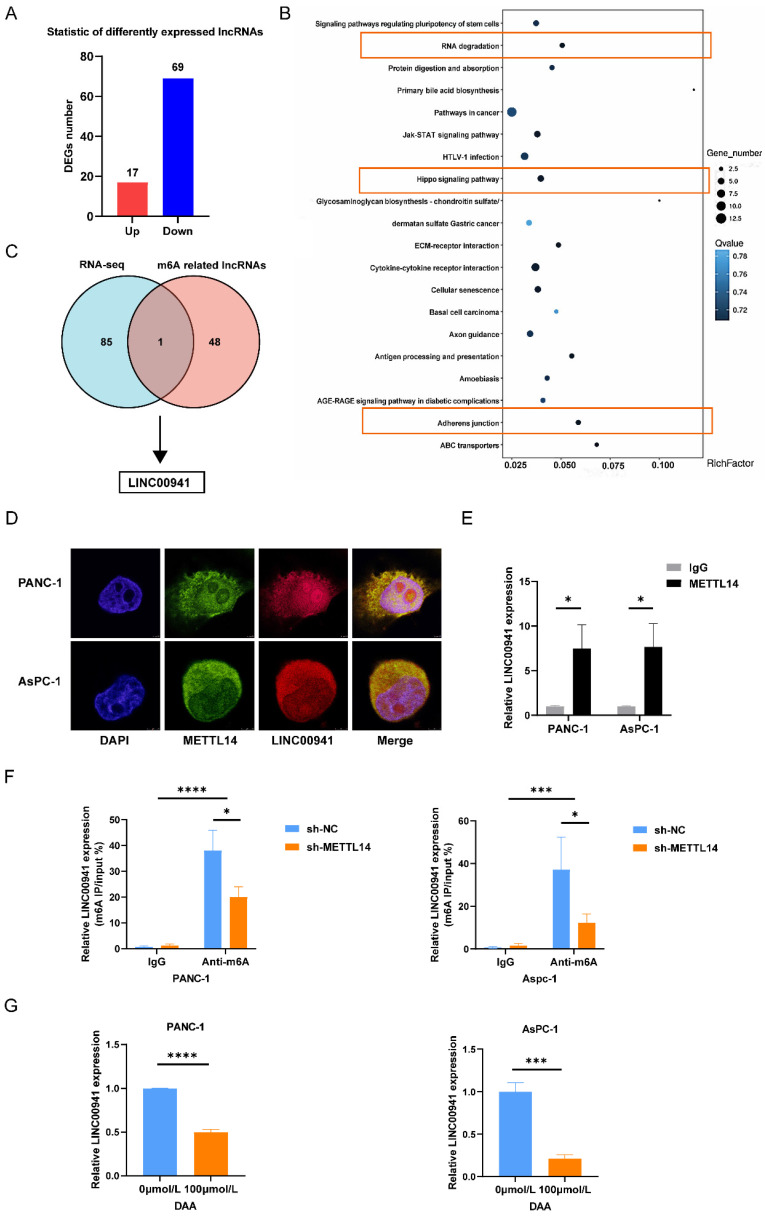
LINC00941 was identified as the downstream target of METTL14. (A) Different expressed lncRNAs were screened out between sh-NC and sh-METTL14 PANC-1 cells by RNA-seq. (B) KEGG analysis of DEGs enrichment. (C) Venn diagram exhibited the overlapped lncRNA between different expressed lncRNAs and m6A-related lncRNAs. (D) IF/Fish assays confirmed the colocalization of METTL14 and LINC00941 in PANC-1 and AsPC-1 cells. (E) RNA immunoprecipitation of METTL14 and LINC00941. (F) METTL14 knockdown decreased the m6A level of LINC00941 in PANC-1 and AsPC-1 cells. (G) DAA inhibited the expression level of LINC00941. * *p* < 0.05, ** *p* < 0.01, *** *p* < 0.001, **** *p* < 0.0001.

To explore the potential mechanisms of METTL14, we performed RNA-seq in sh-NC and sh-METTL14 pancreatic cancer cells (PANC-1). After screening, we observed that 17 lncRNAs were upregulated and 69 lncRNAs were downregulated (fold change >1.0 and *p* < 0.05) (Figure 4A). KEGG analysis indicated that the differentially expressed mRNAs/lncRNAs were mainly enriched in the pathways of adherens junction, Hippo signaling pathway and RNA degradation (Figure 4B), which were closely related to the metastasis of cancer 23-25. To further investigate the lncRNAs that may be modified by m6A, we screened lncRNAs from the TCGA databases via Pearson correlation (|coefficient| > 0.3 and *p* < 0.001) with m6A regulators (Table S4), and finally we obtained 517 m6A-related lncRNAs (Table S5). Forty-nine m6A-related lncRNAs (Table S6) with prognostic value were identified through univariate Cox regression analysis. Then, we compared the 49 m6A related lncRNAs with the differentially expressed lncRNAs identified by RNA-seq analysis. The results showed that LINC00941 was the only lncRNA overlapping between the two groups; therefore, we selected it for further study (Figure 4C). The RT-qPCR results confirmed that the expression of LINC00941 was positively correlated with METTL14 (Figure S4A). RIP and IF/FISH experiments were conducted to evaluate the interaction between METTL14 and LINC00941 (Figure 4D-E). To further verify that LINC00941 underwent METTL14-mediated m6A modification, we performed MeRIP-PCR on PC cell lines, and the results were in line with expectations. METTL14 knockdown decreased the m6A level of LINC00941 in PC cells (Figure 4F). In contrast, METTL14 overexpression increased m6A modification of LINC00941 in PC cells (Figure S4B). Furthermore, we treated PC cells with DAA, an m6A methylation inhibitor and found that the expression of LINC00941 was lower than that in untreated cells (Figure 4G). The above conclusions confirmed that LINC00941 is a downstream target and that the m6A methylation of LINC00941 is meditated by METTL14 directly.

### METTL14 promoted PC metastasis by targeting LINC00941

**Figure 5:**
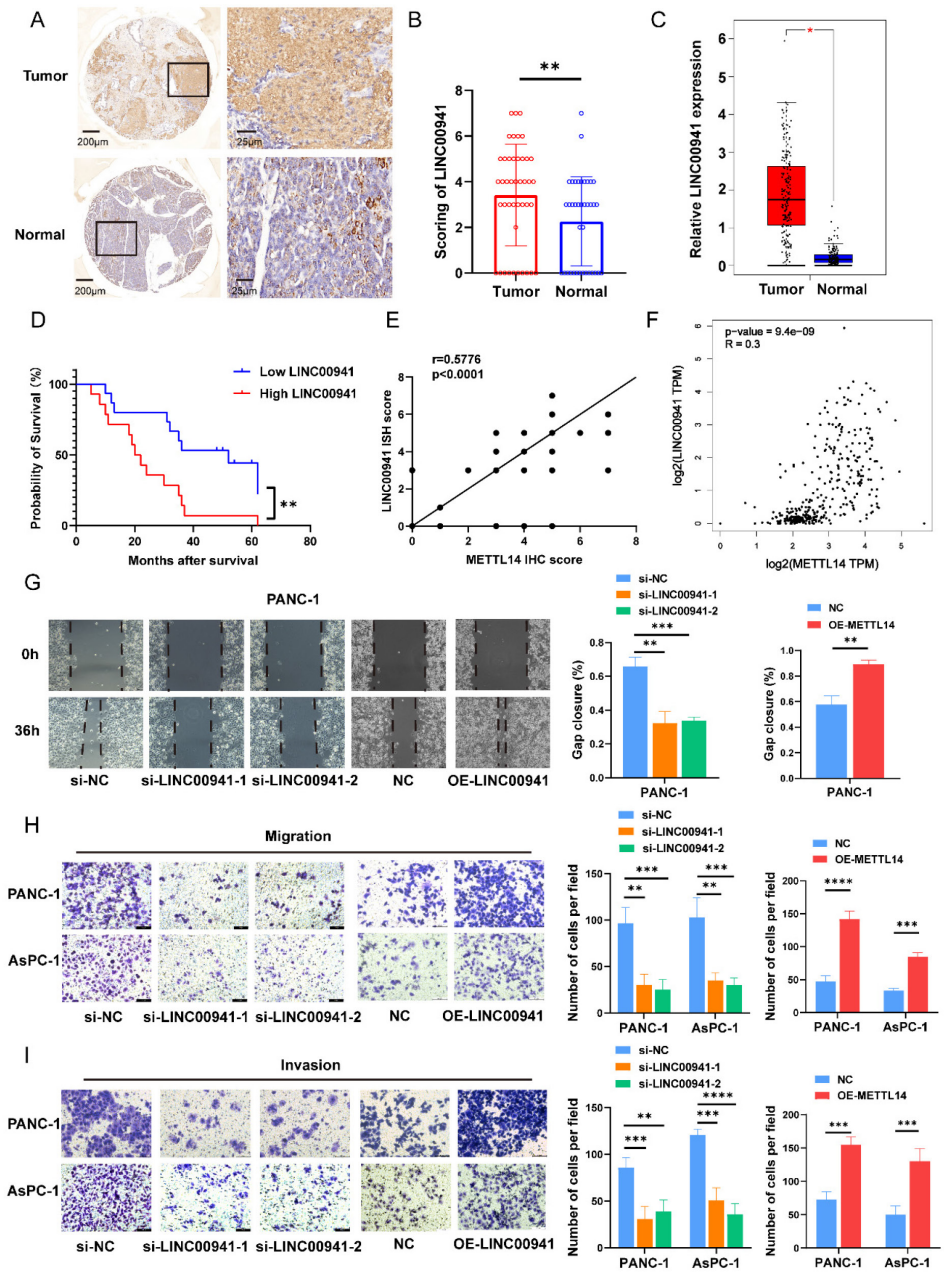
LINC00941 was associated with poor prognostics in PC patients and promoted PC cell migration and invasion. (A) ISH staining results of LINC00941 expression in PC and para-carcinoma tissue (Left image, scale bar, 200 μm; magnification, 50×; right image, scale bar, 50 μm; magnification, 400×). (B) ISH scoring of 42 pairs of tumor and adjacent tissues in PC patients. (C) Expression level of LINC00941 between tumor and normal tissues in TCGA-GTEx. (D) The survival time of PC patients was associated with the expression of LINC00941 of TMA. (E) The correlation between the scores of LINC00941 and METTL14 in TMA. (F) The correlation between LINC00941 and METTL14 in TCGA database. (G) The migration abilities of LINC00941 knockdown and overexpression in PANC-1 cells were measured using wound-healing assays. (H-I) The migration and invasion abilities of LINC00941 knockdown and overexpression in PANC-1 and AsPC-1 cells were measured using transwell assays. * *p* < 0.05, ** *p* < 0.01, *** *p* < 0.001, **** *p* < 0.0001.

First, we measured the expression of LINC00941 in PC patients. In situ hybridization was performed on 42 pairs of tumor and para-carcinoma tissues using the LINC00941 probe. The results of staining showed that the enrichment of LINC00941 in the tumor tissues was higher than that in the paracarcinoma tissues (Figure 5A-B). This finding was confirmed in the TCGA and GTEx databases (Figure 5C). The survival curve indicated that higher expression of LINC00941 led to a shorter survival time (Figure 5D). Moreover, according to the scoring results, higher expression of LINC00941 was associated with higher AJCC stages and N stages in PC patients (Figure S5A-B, Table S7), consistent with the pattern of METTL14 described above. Correlation analysis of staining scores showed that LINC00941 expression was significantly correlated with METTL14 expression at the tissue level (Figure 5E, Figure S5C), which was also confirmed in the TCGA and GTEx databases (Figure 5F). Next, wound healing and Transwell assays were performed to reveal the role of LINC00941 in the metastasis of pancreatic cancer. Both gain- and loss-of-function studies were conducted after knockout and overexpression efficiency verification (Figure S5D). The wound-healing assay results indicated that LINC00941 depletion reduced the migration speed of PANC-1 cells but forced expression of LINC00941 promoted their motility (Figure 5G). As expected, the same trend was verified by Transwell migration and Matrigel invasion assays (Figure 5H-I). In additional, we obtain similar results in AsPC-1 cells (Figure 5H-I, Figure S5E). In vivo studies demonstrated that knockout of LINC00941 the inhibited metastasis of pancreatic cancer (Figure S5F-G).

**Figure 6:**
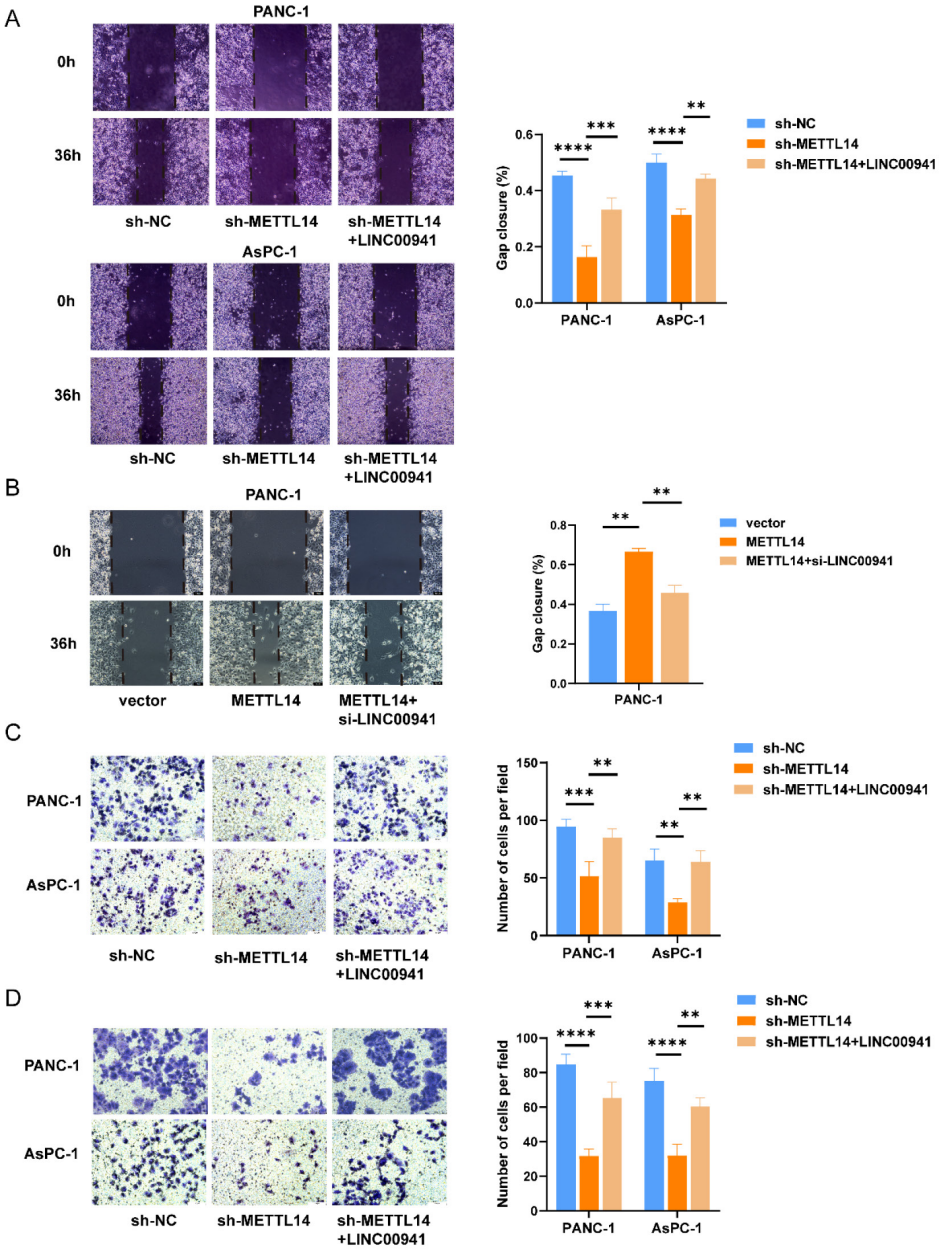
METTL14 promoted PC metastasis by targeting LINC00941. (A) Overexpression of LINC00941 rescued the impaired migration abilities of METTL14 knockdown PC cells in wound-healing assays. (B) Inhibition of LINC00941 could reduce the promoting effects of METTL14 on PANC-1 cell migration measured by wound-healing assays. (C-D) Overexpression of LINC00941 rescued the impaired migration and invasion abilities of METTL14 knockdown PC cells in transwell assays. * *p* < 0.05, ** *p* < 0.01, *** *p* < 0.001, **** *p* < 0.0001.

To further explore the regulatory effect of METTL14 on LINC00941, rescue experiments were conducted. As expected, the results showed that overexpression of LINC00941 rescued the impaired migration and invasion abilities of METTL14 knockdown PC cells (Figure 6A, C-D). Furthermore, inhibition of LINC00941 reduced the promoting effects of METTL14 on cell migration and invasion (Figure 6B, Figure S6A-C). The above results demonstrated that METTL14 could regulate the biological function of PC cells by targeting LINC00941.

### METTL14 enhanced LINC00941 stability through an m6A-IGF2BP2-dependent manner

**Figure 7:**
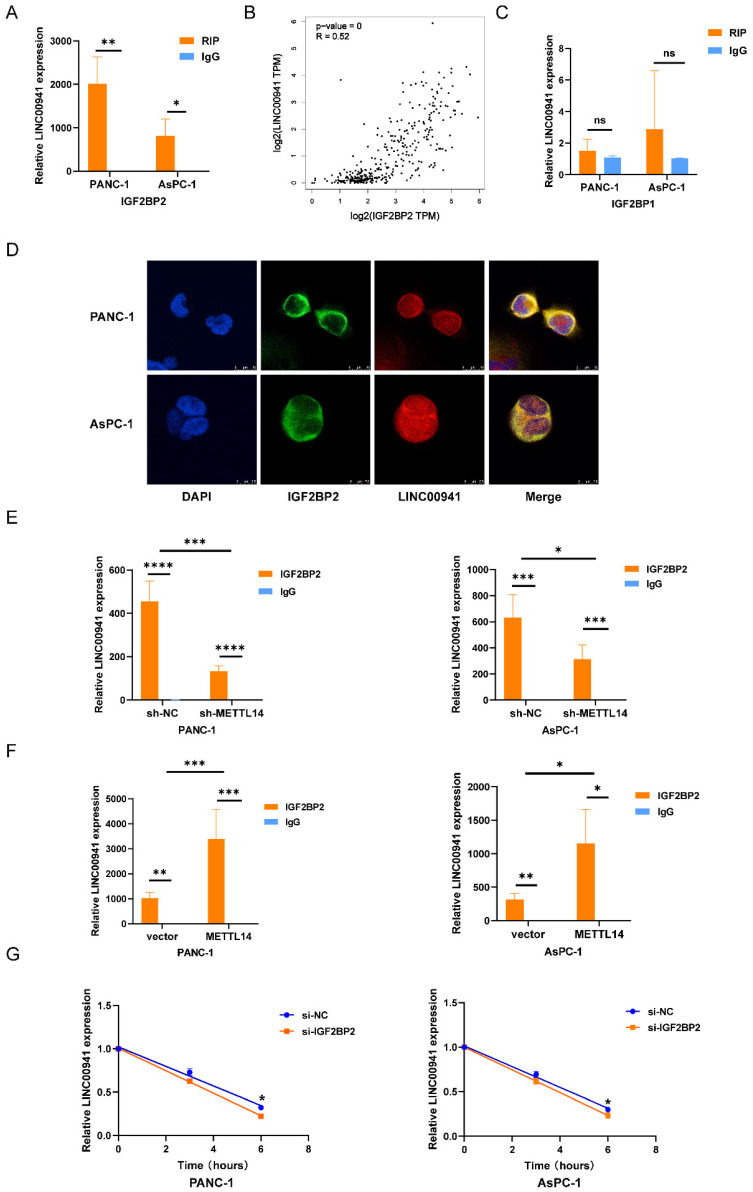
LINC00941 stability was enhanced by METTL14 through a m6A-IGF2BP2-dependent manner. (A) RNA immunoprecipitation of IGF2BP2 and LINC00941. (B) The correlation between LINC00941 and IGF2BP2 in TCGA database. (C) RNA immunoprecipitation of IGF2BP1 and LINC00941. (D) IF/Fish assays confirmed the colocalization of IGF2BP2 and LINC00941 in PANC-1 and AsPC-1 cells. (E) Knockdown of METTL14 weaken the combination of IGF2BP2 and LINC00941. (F) Overexpression of METTL14 enhanced the binding capacity of IGF2BP2 and LINC00941. (G) Knockdown of IGF2BP2 reduced the stability of LINC00941. * *p* < 0.05, ** *p* < 0.01, *** *p* < 0.001, **** *p* < 0.0001.

m6A methylation is a labeling mechanism that needs to be recognized by m6A "reader" proteins for the subsequent processing of target RNAs. To uncover the recognition mechanism, we identified a potential methylation reader that might interact with m6A-modified LINC00941 through the ENCORI database 26. IGF2BP1 and IGF2BP2 were described as potential readers (Table S8). Subsequently, we conducted an RIP assay to verify the above readers. The RIP assay results showed much more binding of LINC00941 in PANC-1 and AsPC-1 cells treated with an antibody against IGF2BP2 than in those treated with IgG (Figure 7A). In addition, the expression of IGF2BP2 and LINC00941 was shown to be positively correlated in the TCGA database for PC, as shown in Figure 7B, suggesting a potential positive regulatory mechanism. However, the results did not differ between the IGF2BP1 and IgG groups in PANC-1 and AsPC-1 cells (Figure 7C). Then, an IF/FISH assay was performed in PANC-1 and AsPC-1 cells to visualize the localization of IGF2BP2 and LINC00941. As shown in Figure 7D, IGF2BP2 and LINC00941 were colocalized in both cytoplasm and nucleus. Moreover, the binding capacity between IGF2BP2 and LINC00941 was weakened when METTL14 was knocked down (Figure 7E). Recognition of LINC00941 by IGF2BP2 was more robust in METTL14-overexpressing PC cells (Figure 7F). To further explore the mechanism, IGF2BP2 was knock down (Figure S7A-B), and we chose si-IGF2BP2-2, with the higher knockdown efficiency, for the follow-up experiment. We discovered that the degradation rate of LINC00941 was accelerated as IGF2BP2 was knock down, suggesting that recognition by IGF2BP2 can promote the RNA stability of LINC00941 (Figure 7G). Therefore, our data illustrated that the methylated LINC00941 was directly recognized by IGF2BP2 and that the binding capacity of IGF2BP2 and LINC00941 could be increased by METTL14, which contributes to the inhibition of RNA degradation. In summary, METTL14 enhanced LINC00941 stability through an m6A-IGF2BP2-dependent pathway.

## Discussion

Over the past several decades, pancreatic cancer has drastically increased in prevalence worldwide and is predicted to continue to be the main cause of cancer-related death 27. Although inherited genetic variables cannot be changed directly, they have a significant role in pancreatic cancer risk. In addition to shedding light on the etiology of pancreatic cancer, the identification of the genetic modification that causes this disease offers the chance to direct early detection efforts.

In recent years, m6A methylation modification has received considerable attention due to its ubiquitous dysregulation in many diseases 28. As a central component of the N6-methytransferase complex, METTL14 has been implicated in a variety of cancers, including gastric cancer 29, colorectal cancer 30, 31, hepatocellular cancer 32 and other types of cancers. Interestingly, the biological role of METTL14 in different tumors is variable.

This variability may be explained by the tissue-specific expression of METTL14. In this work, we discovered that METTL14 expression was markedly elevated in PC tissues and that increased METTL14 expression contribute to worse prognosis in PC patients. To further elucidate the effect of METT14 on the migration and invasion of PC cells, we performed in vitro and in vivo experiments. We found that silencing METTL14 inhibited the migration and invasion of PC cells. Next, we conducted RNA-seq in PC cells with METTL14 knockdown to further explore the mechanism underlying this phenomenon. Combining these results with the data from TCGA analysis, we focused on LINC00941 as the downstream target of METTL14. We verified that LINC00941 was overexpressed in PC tissues and found that it was associated with poor prognosis. The result of MeRIP and RIP assays confirmed that LINC00941 was regulated by METTL14 in an m6A-dependent manner. In vitro and in vivo experiments indicated LINC00941 was involved in the migration and invasion of PC cells. In addition, the decreases in the invasion and migration abilities caused by METTL14 knockdown were rescued by overexpression of LINC00941 in PC cells. Previous studies 33 have reported that m6A binding proteins can recognize the m6A modification to regulate different downstream biological functions. IGF2BP2, a member of the insulin-like growth factor 2 mRNA-binding protein family, was selected as the specific m6A reader of LINC00941. Our research also proved that METTL14 had the ability to regulate the interaction between IGF2BP2 and LINC00941. The interaction was impaired upon METTL14 knockdown, indicating that m6A modification of LINC00941 was the key determinant of its binding to IGF2BP2. Moreover, IGF2BP2 maintained LINC00941 stability by recognizing m6A-modified LINC00941 to promote the metastasis of PC. Overall, these findings provide a novel epigenetic component to augment our understanding of the pathophysiology in PC. METTL14 might serve as a prognostic marker and therapeutic target of PC.

Previous studies have shown that m6A modification cannot not only affects mRNAs, but also influences the synthesis and function of noncoding RNAs, such as lncRNAs 34. m6A modifications influence the RNA‒DNA structure and modulate the interactions between lncRNAs and specific DNA sites. In addition, m6A modification creates binding sites for m6A reader proteins or alters the local structure of RNAs, inducing the binding of RBPs to regulate the function of these lncRNAs 35. Cui et al. 36 found that FTO removes m6A modifications from LINC00022, thereby inhibiting its degradation mediated by the m6A reader YTHDF2, which contributes to the cell proliferation-promoting effects of FTO-induced LINC00022 in esophageal squamous cell carcinoma. The m6A writer WTAP mediates the methylation of the lncRNA DIAPH1-AS1 and enhances its stability, resulting in the promotion of nasopharyngeal carcinoma growth and metastasis 37. LINC00941, also called lncRNA-MUF, can regulate the biological function and progression of tumors. Recently, investigators have examined the influence of LINC00941 on tumorigenesis in hepatocellular carcinoma 38 and glioblastoma multiforme 39. In addition, LINC00941 promotes epithelial-to-mesenchymal transition and distant metastasis by inhibiting autophagy and cell proliferation in thyroid carcinoma 40.

The IGF2BP2 family is a highly conserved RNA-binding protein family, and high expression levels of IGF2BP family members can be detected during carcinogenesis and are considered to be associated with tumor progression and poor prognosis 41. IGF2BP proteins are composed of tandem arrangements of two KM domains and RNA-recognition motifs (RRMs) located in the C-terminal and N-terminal regions, respectively 42. According to in vitro research 43, 44, RNA-binding is mainly accomplished via the KH domains, while the stability of the IGF2BP-RNA complex is maintained by the RRMs. IGF2BP2 preferentially bound to m6A-modified RNAs, since the results showed that IGF2BP2 had a threefold higher affinity for m6A-modified RNAs than for unmodified RNAs 45. In addition to mRNAs, noncoding RNAs with m6A modification can also interact with and be stabilized by IGF2BP2 46. More importantly, our study confirmed that LINC00941 can be recognized by IGF2BP2 and that METTL14 can affect this interaction. These findings broaden our mechanistic understanding.

## Conclusion

In summary, our research elucidated the crucial role of METTL14-mediated m6A modification in PC metastasis and revealed an m6A-dependent regulatory mechanism. METTL14 and LINC00941 can promote the migration and invasion of PC cells. Mechanistically, by methylating LINC00941, METTL14 promoted the recognition of LINC00941 by IGF2BP2, thereby stabilizing LINC00941 and promoting the migration and invasion of PC cells. Based on these findings, we deduced that the METTL14-LINC00941-IGF2BP2 axis might be a potential treatment target in PC.

## Supplementary Material

Supplementary figures and tables.

## Figures and Tables

**Table 1:** Correlations between METTL14 and clinicopathologic parameters in PC patients

Parameters	No. (n=42)	METTL14 expression		χ^2^	P
		high	low		
Gender					
Male	25	11	14	0.3183	0.5726
Female	17	6	11		
Age (years)					
≤65	24	6	18	5.567	0.0183*
>65	18	11	7		
Tumor location					
Head	34	11	23	4.889	0.027*
Body/tail	8	6	2		
Tumor size (cm)					
≤2	8	3	5	0.03633	0.8488
>2	34	14	20		
Pathologic grade					
Ⅰ-Ⅱ	22	9	13	0.003594	0.9522
Ⅲ	20	8	12		
N stage					
N0	28	7	21	8.351	0.0039**
Nx	14	10	4		
Perineural invasion					
Absent	10	5	5	0.4941	0.4821
Present	32	12	20		
AJCC stage					
Ⅰ	25	5	20	10.75	0.001**
Ⅱ-Ⅳ	17	12	5		

(The *p* value was calculated by chi-squared analysis) * *p* < 0.05; ** *p* < 0.01

## References

[1] Mizrahi JD, Surana R, Valle JW, Shroff RT (2020). Pancreatic cancer. Lancet.

[2] Siegel RL, Miller KD, Fuchs HE, Jemal A (2022). Cancer statistics, 2022. CA Cancer J Clin.

[3] Neoptolemos JP, Kleeff J, Michl P, Costello E, Greenhalf W, Palmer DH (2018). Therapeutic developments in pancreatic cancer: current and future perspectives. Nat Rev Gastroenterol Hepatol.

[4] Jiang X, Liu B, Nie Z, Duan L, Xiong Q, Jin Z (2021). The role of m6A modification in the biological functions and diseases. Signal Transduct Target Ther.

[5] Fang Z, Mei W, Qu C, Lu J, Shang L, Cao F (2022). Role of m6A writers, erasers and readers in cancer. Exp Hematol Oncol.

[6] Zaccara S, Ries RJ, Jaffrey SR (2019). Reading, writing and erasing mRNA methylation. Nat Rev Mol Cell Biol.

[7] Li X, Ma S, Deng Y, Yi P, Yu J (2022). Targeting the RNA m(6)A modification for cancer immunotherapy. Mol Cancer.

[8] Wang K, Liu H, Hu Q, Wang L, Liu J, Zheng Z (2022). Epigenetic regulation of aging: implications for interventions of aging and diseases. Signal Transduct Target Ther.

[9] Boulias K, Greer EL (2022). Biological roles of adenine methylation in RNA. Nat Rev Genet.

[10] Zhang Z, Wang L, Zhao L, Wang Q, Yang C, Zhang M (2022). N6-methyladenosine demethylase ALKBH5 suppresses colorectal cancer progression potentially by decreasing PHF20 mRNA methylation. Clin Transl Med.

[11] Lin Z, Wan AH, Sun L, Liang H, Niu Y, Deng Y (2023). N6-methyladenosine demethylase FTO enhances chemo-resistance in colorectal cancer through SIVA1-mediated apoptosis. Mol Ther.

[12] Liu HT, Zou YX, Zhu WJ, Sen L, Zhang GH, Ma RR (2022). lncRNA THAP7-AS1, transcriptionally activated by SP1 and post-transcriptionally stabilized by METTL3-mediated m6A modification, exerts oncogenic properties by improving CUL4B entry into the nucleus. Cell Death Differ.

[13] Chang YZ, Chai RC, Pang B, Chang X, An SY, Zhang KN (2021). METTL3 enhances the stability of MALAT1 with the assistance of HuR via m6A modification and activates NF-kappaB to promote the malignant progression of IDH-wildtype glioma. Cancer Lett.

[14] Zhang Y, Liu X, Wang Y, Lai S, Wang Z, Yang Y (2022). The m(6)A demethylase ALKBH5-mediated upregulation of DDIT4-AS1 maintains pancreatic cancer stemness and suppresses chemosensitivity by activating the mTOR pathway. Mol Cancer.

[15] Liu SJ, Dang HX, Lim DA, Feng FY, Maher CA (2021). Long noncoding RNAs in cancer metastasis. Nat Rev Cancer.

[16] Tan YT, Lin JF, Li T, Li JJ, Xu RH, Ju HQ (2021). LncRNA-mediated posttranslational modifications and reprogramming of energy metabolism in cancer. Cancer Commun (Lond).

[17] Peng WX, Koirala P, Mo YY (2017). LncRNA-mediated regulation of cell signaling in cancer. Oncogene.

[18] McCabe EM, Rasmussen TP (2021). lncRNA involvement in cancer stem cell function and epithelial-mesenchymal transitions. Semin Cancer Biol.

[19] Ai Y, Wu S, Zou C, Wei H (2020). LINC00941 promotes oral squamous cell carcinoma progression via activating CAPRIN2 and canonical WNT/beta-catenin signaling pathway. J Cell Mol Med.

[20] Wang J, He Z, Liu X, Xu J, Jiang X, Quan G (2022). LINC00941 promotes pancreatic cancer malignancy by interacting with ANXA2 and suppressing NEDD4L-mediated degradation of ANXA2. Cell Death Dis.

[21] Wu N, Jiang M, Liu H, Chu Y, Wang D, Cao J (2021). LINC00941 promotes CRC metastasis through preventing SMAD4 protein degradation and activating the TGF-beta/SMAD2/3 signaling pathway. Cell Death Differ.

[22] Xu M, Cui R, Ye L, Wang Y, Wang X, Zhang Q (2021). LINC00941 promotes glycolysis in pancreatic cancer by modulating the Hippo pathway. Mol Ther Nucleic Acids.

[23] Kim J (2022). Cell Dissemination in Pancreatic Cancer. Cells.

[24] Zinatizadeh MR, Miri SR, Zarandi PK, Chalbatani GM, Raposo C, Mirzaei HR (2021). The Hippo Tumor Suppressor Pathway (YAP/TAZ/TEAD/MST/LATS) and EGFR-RAS-RAF-MEK in cancer metastasis. Genes Dis.

[25] Chen J, Wu Y, Luo X, Jin D, Zhou W, Ju Z (2021). Circular RNA circRHOBTB3 represses metastasis by regulating the HuR-mediated mRNA stability of PTBP1 in colorectal cancer. Theranostics.

[26] Li JH, Liu S, Zhou H, Qu LH, Yang JH (2014). starBase v2.0: decoding miRNA-ceRNA, miRNA-ncRNA and protein-RNA interaction networks from large-scale CLIP-Seq data. Nucleic Acids Res.

[27] Klein AP (2021). Pancreatic cancer epidemiology: understanding the role of lifestyle and inherited risk factors. Nat Rev Gastroenterol Hepatol.

[28] Mathiyalagan P, Adamiak M, Mayourian J, Sassi Y, Liang Y, Agarwal N (2019). FTO-Dependent N(6)-Methyladenosine Regulates Cardiac Function During Remodeling and Repair. Circulation.

[29] Fan HN, Chen ZY, Chen XY, Chen M, Yi YC, Zhu JS (2022). METTL14-mediated m(6)A modification of circORC5 suppresses gastric cancer progression by regulating miR-30c-2-3p/AKT1S1 axis. Mol Cancer.

[30] Yang X, Zhang S, He C, Xue P, Zhang L, He Z (2020). METTL14 suppresses proliferation and metastasis of colorectal cancer by down-regulating oncogenic long non-coding RNA XIST. Mol Cancer.

[31] Chen X, Xu M, Xu X, Zeng K, Liu X, Pan B (2020). METTL14-mediated N6-methyladenosine modification of SOX4 mRNA inhibits tumor metastasis in colorectal cancer. Mol Cancer.

[32] Du L, Li Y, Kang M, Feng M, Ren Y, Dai H (2021). USP48 Is Upregulated by Mettl14 to Attenuate Hepatocellular Carcinoma via Regulating SIRT6 Stabilization. Cancer Res.

[33] Wang T, Kong S, Tao M, Ju S (2020). The potential role of RNA N6-methyladenosine in Cancer progression. Mol Cancer.

[34] Hou P, Meng S, Li M, Lin T, Chu S, Li Z (2021). LINC00460/DHX9/IGF2BP2 complex promotes colorectal cancer proliferation and metastasis by mediating HMGA1 mRNA stability depending on m6A modification. J Exp Clin Cancer Res.

[35] Huang X, Guo H, Wang L, Yang L, Shao Z, Zhang W (2022). Recent advances in crosstalk between N6-methyladenosine (m6A) modification and circular RNAs in cancer. Mol Ther Nucleic Acids.

[36] Cui Y, Zhang C, Ma S, Li Z, Wang W, Li Y (2021). RNA m6A demethylase FTO-mediated epigenetic up-regulation of LINC00022 promotes tumorigenesis in esophageal squamous cell carcinoma. J Exp Clin Cancer Res.

[37] Li ZX, Zheng ZQ, Yang PY, Lin L, Zhou GQ, Lv JW (2022). WTAP-mediated m(6)A modification of lncRNA DIAPH1-AS1 enhances its stability to facilitate nasopharyngeal carcinoma growth and metastasis. Cell Death Differ.

[38] Yan X, Zhang D, Wu W, Wu S, Qian J, Hao Y (2017). Mesenchymal Stem Cells Promote Hepatocarcinogenesis via lncRNA-MUF Interaction with ANXA2 and miR-34a. Cancer Res.

[39] Shree B, Tripathi S, Sharma V (2021). Transforming Growth Factor-Beta-Regulated LncRNA-MUF Promotes Invasion by Modulating the miR-34a Snail1 Axis in Glioblastoma Multiforme. Front Oncol.

[40] Gugnoni M, Manicardi V, Torricelli F, Sauta E, Bellazzi R, Manzotti G (2021). Linc00941 Is a Novel Transforming Growth Factor beta Target That Primes Papillary Thyroid Cancer Metastatic Behavior by Regulating the Expression of Cadherin 6. Thyroid.

[41] Yaniv K, Yisraeli JK (2002). The involvement of a conserved family of RNA binding proteins in embryonic development and carcinogenesis. Gene.

[42] Degrauwe N, Suva ML, Janiszewska M, Riggi N, Stamenkovic I (2016). IMPs: an RNA-binding protein family that provides a link between stem cell maintenance in normal development and cancer. Genes Dev.

[43] Wachter K, Kohn M, Stohr N, Huttelmaier S (2013). Subcellular localization and RNP formation of IGF2BPs (IGF2 mRNA-binding proteins) is modulated by distinct RNA-binding domains. Biol Chem.

[44] Aguilar-Garrido P, Otero-Sobrino A, Navarro-Aguadero MA, Velasco-Estevez M, Gallardo M (2022). The Role of RNA-Binding Proteins in Hematological Malignancies. Int J Mol Sci.

[45] Huang H, Weng H, Sun W, Qin X, Shi H, Wu H (2018). Recognition of RNA N(6)-methyladenosine by IGF2BP proteins enhances mRNA stability and translation. Nat Cell Biol.

[46] Hu X, Peng WX, Zhou H, Jiang J, Zhou X, Huang D (2020). IGF2BP2 regulates DANCR by serving as an N6-methyladenosine reader. Cell Death Differ.

