# Supplementary material for: METTL14 Facilitates the Metastasis of Pancreatic Carcinoma by Stabilizing LINC00941 in an m6A-IGF2BP2-Dependent Manner
Source: J Cancer. 2023 Apr 17;14(7):1117–31. doi: 10.7150/jca.84070 (PMC10197944; doi:10.7150/jca.84070)
Supplement: Supplementary file 1 — Supplementary figures and tables. [file jcav14p1117s1.pdf]

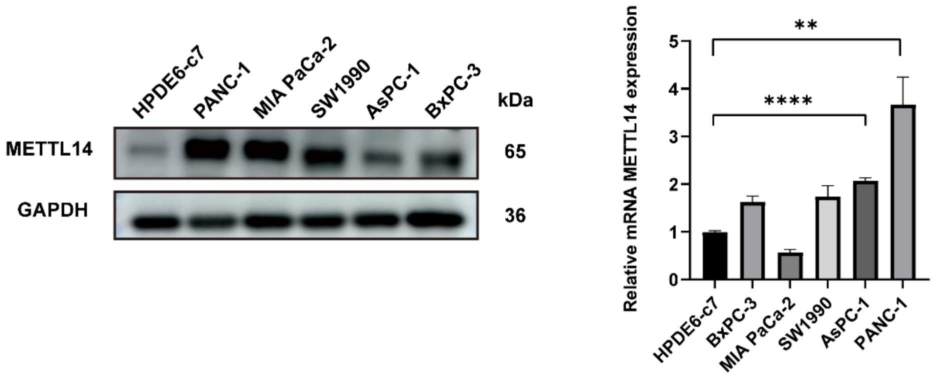

Figure S1

The mRNA and protein levels of METTL14 in PC cell lines and normal pancreatic epithelial cells. * $p < 0.05$, ** $p < 0.01$, *** $p < 0.001$, **** $p < 0.0001$.

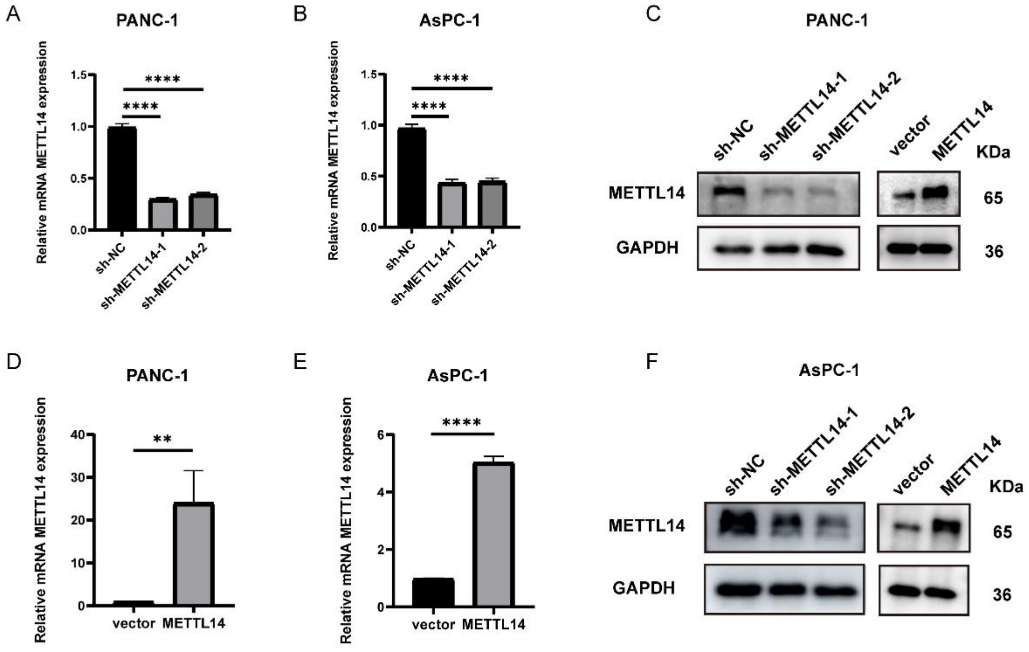

Figure S2

(A-B, D-E) The efficacy of knockdown and overexpression METTL14 in PANC-1 and AsPC-1 cells measured by RT-qPCR. (C, F) The efficacy of knockdown and overexpression METTL14 in PANC-1 and AsPC-1 cells measured through western blot. * $p < 0.05$, ** $p < 0.01$, *** $p < 0.001$, **** $p < 0.0001$.

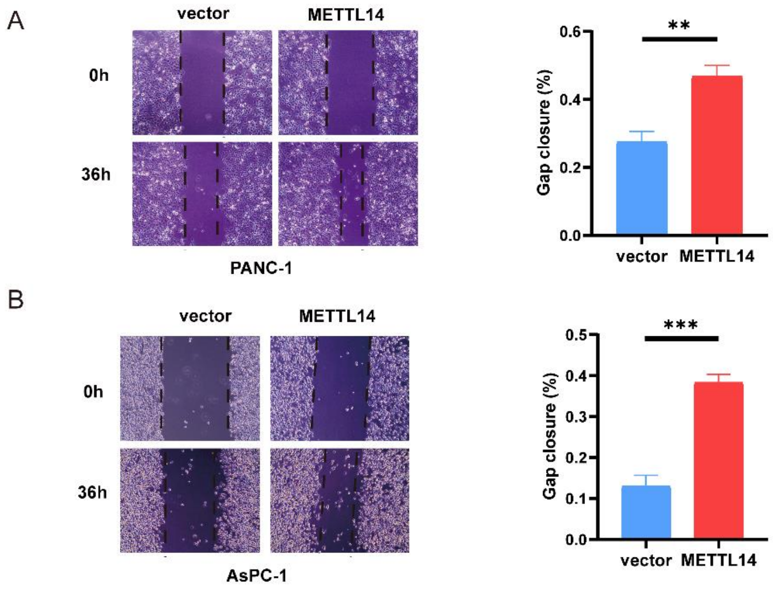

Figure S3
(A-B) Overexpressed METTL14 promoted the migration of PANC-1 and AsPC-1 cells measured by wound-healing assays. * $p < 0.05$, ** $p < 0.01$, *** $p < 0.001$.

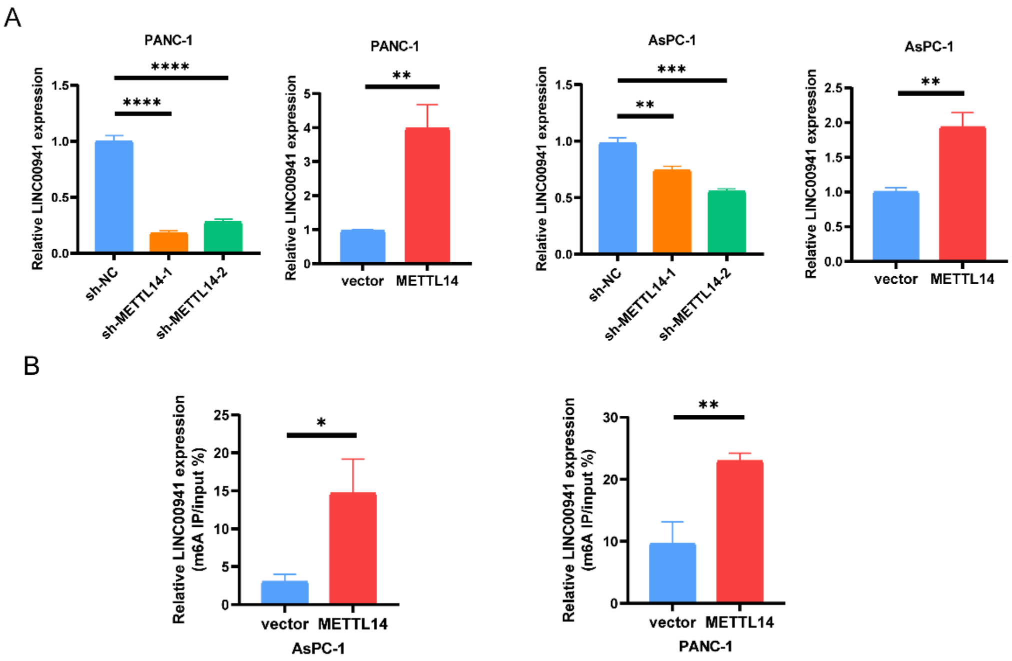

Figure S4
LINC00941 was the downstream target of METTL14. (A) The expression of LINC00941 was positively correlated with METTL14 detected by RT-qPCR. (B) METTL14 overexpression increased the m6A level of LINC00941 in PANC-1 and AsPC-1 cells. * $p < 0.05$, ** $p < 0.01$, *** $p < 0.001$, **** $p < 0.0001$.

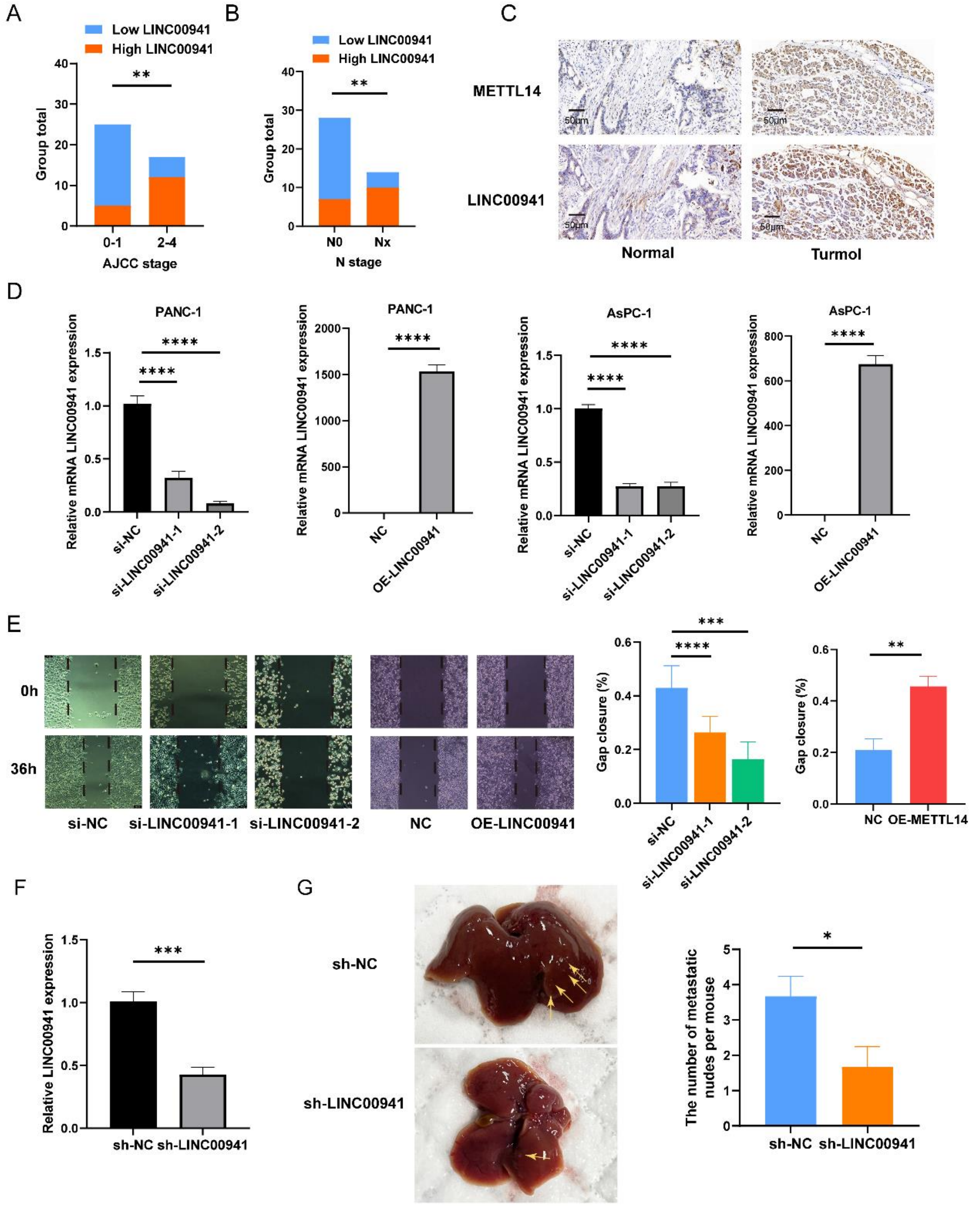

Figure S5

LINC00941 promoted the metastasis of PC cells. (A) AJCC stage was associated with the expression of LINC00941 in PC patients. (B) The lymph node metastasis was associated with level of LINC00941 in PC patients. (C) LINC00941 was significantly correlated with METTL14 at the tissue level (scale bar, 50 μm; magnification, 200×). (D) The efficacy of knockdown and overexpression LINC00941 in PANC-1 and AsPC-1 cells measured by RT-qPCR. (E) The migration abilities of LINC00941 knockdown and overexpression in AsPC-1 cells were measured using wound-healing assays. (F) The efficacy of stable knockdown LINC00941 in PANC-1 cells measured by RT-qPCR. (G) Representative images of metastatic sites in the liver of nude mice

injected with sh-NC or sh-LINC00941 cells. * $p < 0.05$, ** $p < 0.01$, *** $p < 0.001$, **** $p < 0.0001$.

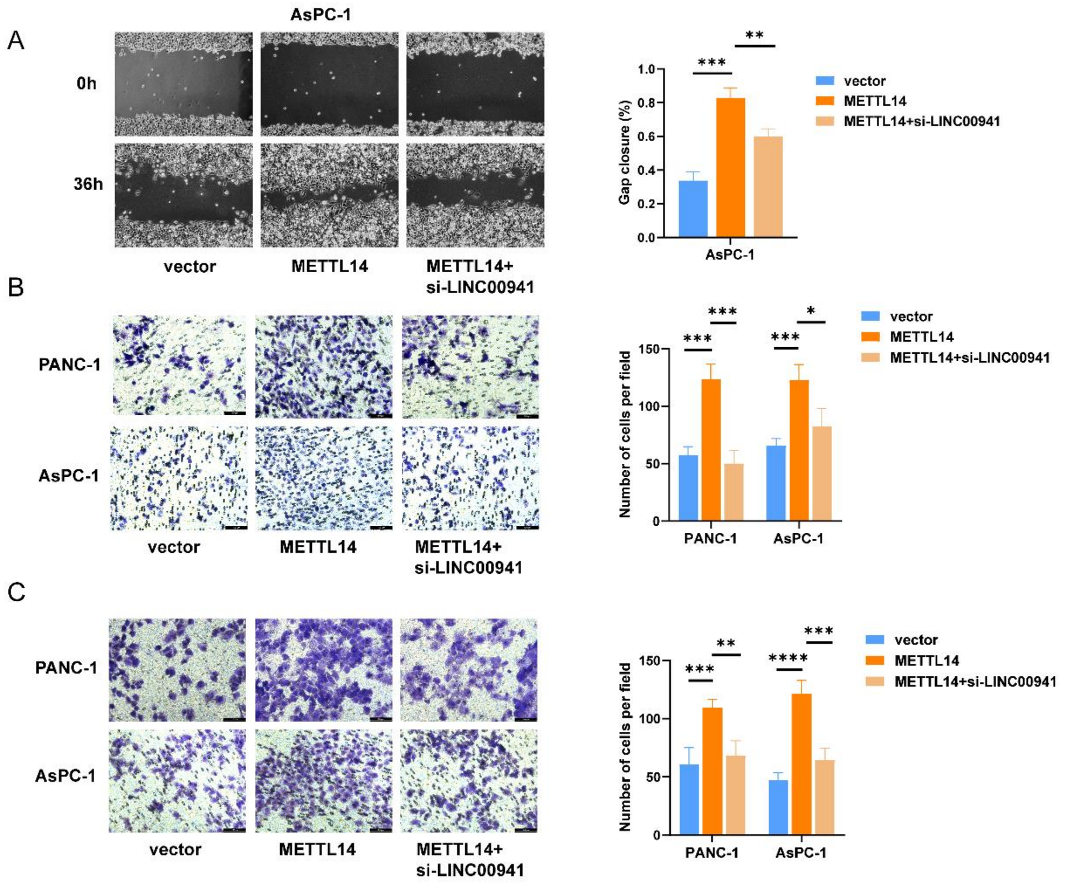

Figure S6

METTL14 promoted PC metastasis by targeting LINC00941. (A) Inhibition of LINC00941 could reduce the promoting effect of METTL14 on AsPC-1 cell migration measured by wound-healing assays. (B-C) Inhibition of LINC00941 could reduce the promoting effect of METTL14 on cell migration and invasion measured by Transwell assays. * $p < 0.05$, ** $p < 0.01$, *** $p < 0.001$, **** $p < 0.0001$.

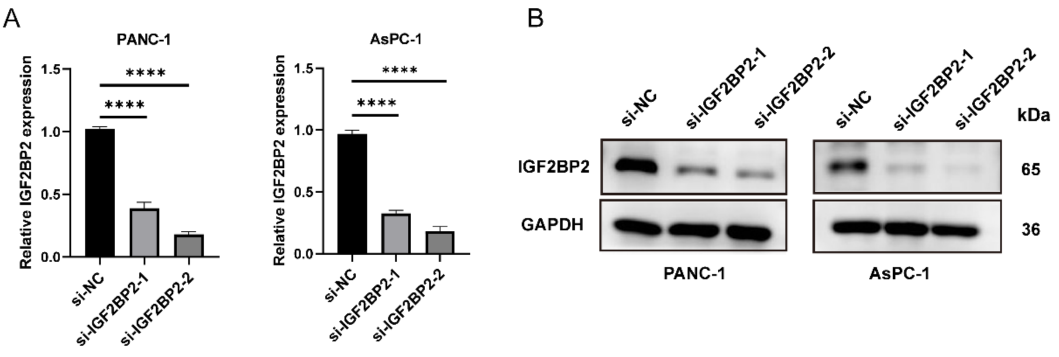

Figure S7

(A-B) The efficacy of knockdown IGF2BP2 in PANC-1 and AsPC-1 cells measured by RT-qPCR and western blot. * $p < 0.05$, ** $p < 0.01$, *** $p < 0.001$, **** $p < 0.0001$.

Table S1 The sequences of siRNA

siRNA	Sequence (5'to3')
METTL14(h)-sh-1	GCTGGACTTGGGATGATATTA
METTL14(h)-sh-2	GGAACAACTCAAGTAGCTTCA
LINC00941(h)-sh-1	CACTACACTCAGCCAAATA
LINC00941(h)-sh-2	GGCATACTGACAATACAAA
LINC00941(h)-si-1	CACTACACTCAGCCAAATA
LINC00941(h)-si-2	GGCATACTGACAATACAAA
IGF2BP2(h)-si-1	GCCGUUGUCAACGUCACAUTT
IGF2BP2(h)-si-2	CCCAGUUUGUUGGUGCCAUTT

Table S2 The primer sequences involved in the research

Gene		Primers (5' to 3')
METTL14	F	CTGAAAGTGCCGACAGCATTGG
	R	CTCTCCTTCATCCAGATACTTACG
GAPDH	F	GTCTCCTCTGACTTCAACAGCG
	R	ACCACCCTGTTGCTGTAGCCAA
LINC00941	F	TAAAGGAGACAGTTGATAGCCAAAC
	R	TGGAGGCTGAGAAGTCCAAGAAC
IGF2BP2	F	GCAGAAAGAGGCAGATGAGACC
	R	TATCTTGGTCCCTGTTTCATGTTC

Table S3 Antibodies involved in the research

Antibodies	Source
METTL14	Proteintech 26158-1-AP
IGF2BP2	Proteintech 11601-1-AP
IGF2BP1	Proteintech 22803-1-AP
IgG	Sangon Biotech D110147-0100

Table S3 m6A Regulators

Gene
METTL3
METTL14
METTL16
VIRMA
RBM15
RBM15B

ZC3H13
WTAP
IGF2BP1
IGF2BP2
IGF2BP3
YTHDC1
YTHDC2
YTHDF1
YTHDF2
YTHDF3
HNRNPA2B1
HNRNPC
RBMX
ALKBH5
FTO

Table S5 517 m6A related lncRNAs

lncRNA
AC087741.2
PSORS1C3
AC009237.15
AC008760.1
AL645608.7
FOXD2-AS1
AP000439.2
MIR100HG
AL022316.1
AP006621.3
AC006042.1
LINC02432
AC012467.1
AP002498.1
AC008608.2
AC027575.2
HOXB-AS3
DGUOK-AS1
AC022034.1
AP005233.2
MINCR
USP30-AS1
AL034397.3
AC009686.2
AC068580.1

AC007114.1
LINC01963
AC106795.2
MID1IP1-AS1
AL109811.2
AP002807.1
AC023043.1
PRRT3-AS1
H19
LINC02041
LINC02381
MAFG-DT
HOTAIRM1
NALT1
SLC25A25-AS1
PIK3CD-AS2
LINC01559
AL031847.1
AC093620.1
MHENCR
AL606834.1
AL445524.1
AC026740.1
AC006942.1
BLACAT1
LINC01836
AC009275.1
AC118754.1
AC104083.1
MNX1-AS1
AP001505.1
MELTF-AS1
AC008443.5
AL121895.2
ZSCAN16-AS1
AC022144.1
EXOC3-AS1
HNF1A-AS1
ITGB1-DT
SNHG17
AC106876.1
AC120498.4
BX470102.1
AC245884.8
AL139246.5

AC124798.1
AC018653.3
AC025154.2
AC010547.2
AC138207.5
LINC02298
AL355338.1
AC010531.6
LINC00578
AL109936.2
AC019117.1
AP006621.4
DLGAP1-AS1
PAXIP1-AS1
AL441992.1
AC108673.3
AL591845.1
AC009065.2
LINC00339
GAS5
LINC00482
DICER1-AS1
MIR4435-2HG
AL049629.1
AF131215.6
SNHG8
THAP9-AS1
AC020558.2
LINC01978
AC104564.3
SPINT1-AS1
LINC01819
ATP6V0E2-AS1
AP006284.1
PCED1B-AS1
C8orf31
RAD51-AS1
AC069281.2
AC245041.1
XXYLT1-AS2
LINC01023
AC093673.1
LINC00346
AC009237.14
AC138696.2

SAPCD1-AS1
AL365181.2
ARRDC1-AS1
U62317.1
LINC00987
AC020910.4
AC004687.1
AL691482.3
AC021146.12
SNHG7
NUP50-DT
AL121761.1
EPB41L4A-AS1
AL161421.1
MEG9
LINC00941
ZNF667-AS1
MNX1-AS2
LINC00443
AC068580.3
AP001625.2
PTOV1-AS2
AC009065.5
AL138930.1
CRNDE
AC005076.1
ELN-AS1
AC011472.1
AC026471.4
AL355001.2
AP002360.1
LINC01503
AC084018.1
IPO5P1
MIR22HG
AL139349.1
AC012640.2
AC034243.1
LINC02086
ANKRD10-IT1
ASMTL-AS1
GAS6-AS1
SNHG25
AC105446.1
AC022509.3

AC104986.2
AC016888.1
AL133410.1
AC007541.1
B3GALT5-AS1
AC103691.1
TNRC6C-AS1
AL512274.1
LINC01770
AL353150.1
LINC00106
AL121929.2
AL512413.1
AC110285.6
SMIM2-AS1
AC004918.1
AC018755.4
CEBPA-DT
AL049840.4
AC084117.1
AL118505.1
AL031058.1
AC026471.3
AC116407.2
MIR200CHG
AP002761.4
MIR210HG
AC010735.2
AC027796.4
AC008760.2
AC092718.4
GATA6-AS1
AC129492.1
AL162586.1
AC073896.4
EBLN3P
LINP1
AC097639.1
AC099509.1
AL139287.1
SMIM25
AC099850.3
SLC12A9-AS1
AC012615.1
AC147067.1

AC004816.1
AC010326.3
AC136475.2
LINC01871
AC025181.2
SNHG3
AC018904.1
AC048341.2
LINC01705
AC016735.1
HOXA-AS2
AC090617.5
AC095057.3
AL451165.2
AL136084.3
Z98257.1
UCA1
AC009133.1
LINC01843
AC107952.2
AC132872.1
AL391988.1
AL133371.2
AC090181.2
SNHG6
SNHG12
AC136475.3
AC156455.1
AL132989.1
LINC00992
CASC8
ARHGAP27P1-BPTFP1-KPNA2P3
TM4SF1-AS1
AL390198.1
DBH-AS1
LINC00239
AL645933.2
FLNB-AS1
TRIM52-AS1
LINC00957
LINC01426
SNHG19
ELF3-AS1
SNHG10
AP000695.2

AC004130.1
AC004148.2
BX284668.5
AC069120.1
AC244197.2
KRT7-AS
AL928654.2
MIR222HG
HAND2-AS1
AC092535.4
AC037198.1
AL035071.1
AC012236.1
MIR7-3HG
AC103702.2
AL121832.2
ILF3-DT
AL049836.1
LINC00261
LINC00685
AC025857.2
LINC01004
LINC01480
AC026401.3
LINC01637
AC112491.1
AC027031.2
LINC00342
AL357033.4
AL022328.2
AL139393.2
SNHG9
AL513165.1
AP000757.1
AC087289.1
TGFB2-AS1
TBILA
AC015922.2
AC004540.2
LINC01006
AC105277.1
LINC02562
BX322234.1
AC104964.1
LINC00926

AFAP1-AS1
LINC01116
AC104958.2
AP001453.2
AL591895.1
AL022322.1
AL139246.3
AC016876.1
AC062037.2
BX293535.1
MIR193BHG
SNHG18
AC020659.1
AL035446.1
AC106900.1
DLGAP1-AS2
ZNF710-AS1
AL080317.1
SSTR5-AS1
AGAP2-AS1
LENG8-AS1
NEAT1
LINC01857
AC022784.1
MIR194-2HG
AL390728.6
LINC01146
LINC00511
AC010503.4
AC080037.1
AL133325.3
SH3PXD2A-AS1
AL365181.3
AC103706.1
PSMB8-AS1
AC015912.3
AC060780.1
AC108134.1
PCAT7
TONSL-AS1
AP006621.2
AL158206.1
AC007405.3
AL606489.1
AC021218.1

AC244153.1
AC092171.2
HCG11
AC113346.1
LINC00659
LBX2-AS1
LINC02482
AL731567.1
TRIM31-AS1
AC087588.2
AL590666.2
AC134312.5
HMGA1P4
AC008735.2
SEMA3B-AS1
AL132712.2
AL021707.8
U62317.2
SLCO4A1-AS1
AL049555.1
DIO3OS
APTR
AP003419.3
AP000894.4
LINC02188
AC239868.1
RPARP-AS1
AC107959.3
SYNPR-AS1
HCP5
AC003965.2
AC004264.1
AC026979.2
MEG3
AC002511.2
AL354836.1
AC009283.1
AC010719.1
AL355472.1
VPS9D1-AS1
AC090152.1
AP003068.2
AC023157.3
AC124016.2
AL109613.1

AC011445.2
AC010542.5
AC090515.4
AC010487.2
UNC5B-AS1
TMEM44-AS1
PCAT6
MIAT
AP000695.1
AC009407.1
AL118516.1
AC245041.2
BX322562.1
MAGI2-AS3
AL392172.1
PRR34-AS1
AC015813.1
RUSC1-AS1
AC084036.1
AC003070.1
YTHDF3-AS1
AC129507.2
AC073957.3
SNHG1
AC130456.3
AL117379.1
AC026368.1
AL023284.4
DANCR
AC092171.5
AC254633.1
MALAT1
AC040169.1
AC016957.2
LINC02593
URB1-AS1
AP001033.2
LINC01137
AC008105.3
TNFRSF14-AS1
AL691432.2
AC243960.1
AL355312.4
PRKAG2-AS1
ARHGAP5-AS1

LINC01370
AP003555.3
LINC00857
AC132872.3
DNAJC3-DT
CYTOR
AC138207.2
AC068473.5
NKILA
AL132712.1
AP000892.2
AC133552.5
AC008764.2
LINC01094
AC242842.1
DCST1-AS1
LINC01089
OLMALINC
AL121820.2
AP001189.3
AC020907.1
AC013275.1
CD44-AS1
ITGB2-AS1
LINC00920
LINC01133
AL360181.2
FGF14-AS2
MIR31HG
FOXP4-AS1
AC005840.4
PITPNA-AS1
AC093110.1
AL035661.1
OSER1-DT
AP000757.2
AC090559.1
AC039056.2
PVT1
LINC01315
AL049840.1
KMT2E-AS1
AC004870.2
AL365226.2
TMEM92-AS1

AC245100.7
SNHG4
AC091563.1
AC110285.2
AC015849.3
AL022323.1
ZNF793-AS1
AC098613.1
RNF157-AS1
AC040977.1
LINC01176
TNFRSF10A-AS1
AP003555.2
AL583785.1
AL162171.2
AL021707.6
AL390719.2
AC055822.1
AL133370.1
AC020916.1
LINC02038
SLC9A3-AS1
AC024060.1
AC008610.1
AL513534.1
AC008771.1
AC114488.1
LINC02001
AP001107.9
AC091271.1
WNT5A-AS1
AC002553.2
AC092171.4
HOXB-AS1
AC009041.2
AC105942.1
LINC00524
AF131215.5
AC027117.1
AL355353.1
AC064807.2
CCDC18-AS1

Table S6 Forty-nine m6A related lncRNAs with prognostic value

lncRNA
AP000439.2
AP005233.2
AC023043.1
LINC01559
MHENCR
AL606834.1
AL121895.2
ITGB1-DT
LINC02298
ATP6V0E2-AS1
C8orf31
AC245041.1
U62317.1
LINC00941
PTOV1-AS2
AC009065.5
LINC02086
AC007541.1
AL049840.4
AC099850.3
AC012615.1
AC147067.1
LINC01705
AC095057.3
CASC8
FLNB-AS1
SNHG10
AP000695.2
AC244197.2
AL035071.1
LINC00685
LINC01004
SNHG9
AL513165.1
AC244153.1
AC092171.2
RPARP-AS1
AC107959.3
AC090515.4
AC009407.1
AL118516.1
AC245041.2

TNFRSF14-AS1
LINC00857
DCST1-AS1
LINC01089
LINC01133
TNFRSF10A-AS1
AC008610.1

Table S7 Correlations between LINC00941 and clinicopathologic parameters in pancreatic cancer patients

**Parameters**	**No. （n=42）**	**LINC00941 expression**		χ^2	**P**
		**high**	**low**		
Gender					
Male	25	12	13	1.451	0.2283
Female	17	5	12		
Age(years)					
≤65	24	9	15	0.2059	0.65
> 65	18	8	10		
Tumor location					
Head	34	12	22	1.99	0.1584
Body/tail	8	5	3		
Tumor size(cm)					
≤2	8	3	5	0.03633	0.8488
> 2	34	14	20		
Pathologic grade					
Ⅰ - Ⅱ	22	7	15	1.82	0.1174
Ⅲ	20	10	9		
N stage					
N0	28	7	21	8.351	0.0039**
Nx	14	10	4		
Perineural invasion					
Absent	10	4	6	0.116	0.7334
Present	32	13	25		
AJCC stage					
0- Ⅰ	25	5	20	10.75	0.001**
Ⅱ -Ⅳ	17	12	5		

(The *p* value was calculated by chi-squared analysis)
* $p < 0.05$; ** $p < 0.01$

Table S8 Potential targets of LINC00941

RBP	Gene Name	Gene Type	cluster Num	clipExp Num	pancancer Num
ADAR	LINC00941	lincRNA	4	2	15

EIF4A3	LINC00941	lincRNA	8	1	16
ELAVL1	LINC00941	lincRNA	3	3	14
FBL	LINC00941	lincRNA	2	2	17
FUS	LINC00941	lincRNA	3	3	13
HNRNPK	LINC00941	lincRNA	1	1	15
IGF2BP1	LINC00941	lincRNA	1	1	19
IGF2BP2	LINC00941	lincRNA	2	1	24
KHDRBS2	LINC00941	lincRNA	1	1	15
MOV10	LINC00941	lincRNA	1	1	11
PCBP2	LINC00941	lincRNA	2	1	13
PRPF8	LINC00941	lincRNA	1	1	9
PTBP1	LINC00941	lincRNA	1	1	12
RBM47	LINC00941	lincRNA	2	1	21
SRSF1	LINC00941	lincRNA	2	2	19
TAF15	LINC00941	lincRNA	1	1	13
TARDBP	LINC00941	lincRNA	1	1	17
TIA1	LINC00941	lincRNA	1	1	19
U2AF2	LINC00941	lincRNA	2	1	13
UPF1	LINC00941	lincRNA	17	2	15

Table S9 Clinic parameters of enrolled PC patients

ID	Gender	Age	Tumor location	Perineural invasion	Pathologic grade	Tumor size	N stage	AJCC stage	OS	Die(1) Survive(0)
14-14010	Female	64	head	Present	2	≤2	N0	ⅠA	-	-
14-15012	Male	58	head	Present	2	≤2	N0	ⅠA	18	1
14-15715	Male	61	head	Present	2	≤2	N0	ⅠA	60	0
14-17391	Female	57	head	Present	3	> 2	N0	ⅠB	35	1
14-14542	Male	72	Body/tail	Present	3	> 2	Nx	ⅡB	-	-
14-25152	Female	67	Body/tail	Absent	1-2	≤2	Nx	ⅡB	36	1
14-20614	Female	55	Head	Present	3	> 2	N0	ⅠB	6	1
14-21920	Female	58	Head	Absent	1-2	> 2	N0	ⅠB	32	1
14-15669	Female	66	Head	Present	3	> 2	N0	ⅠB	-	-
14-20314	Male	62	Head	Absent	3	> 2	N0	ⅠB	-	-
14-10164	Male	64	Head	Absent	2	≤2	N0	ⅠA	52	1
14-12819	Male	53	Head	Present	3	≤2	Nx	ⅡB	35	1
14-16748	Female	79	Head	Present	2	> 2	Nx	ⅡB	5	1
14-33544	Female	64	Head	Present	3	> 2	N0	ⅡA	30	1
14-23595	Male	62	Head	Present	2	> 2	N0	ⅠB	31	1
14-33625	Male	68	Body/tail	Present	3	> 2	N0	ⅡA	8	1
14-39435	Female	69	Head	Present	2	> 2	Nx	ⅡB	10	1
14-10072	Female	64	Head	Present	3	> 2	N0	ⅠB	62	0
15-01165	Male	40	Head	Present	3	> 2	Nx	ⅡB	20	1
15-01166	Female	59	Head	Present	2	> 2	N0	ⅠB	54	0

15-2466	Female	49	Head	Present	3	> 2	N0	Ⅰ B	13	1
15-2987	Male	53	Head	Present	3	> 2	Nx	Ⅱ B	11	1
15-6610	Male	84	Head	Absent	2	> 2	Nx	Ⅱ B	19	1
15-7624	Male	49	Head	Present	3	> 2	N0	Ⅰ B	12	1
15-10956	Female	70	Head	Absent	2	> 2	N0	Ⅰ B	62	1
15-10956	Female	70	Head	Absent	2	> 2	N0	Ⅰ B	62	1
15-9518	Male	70	Head	Absent	3	> 2	N0	Ⅰ B	52	0
15-10462	Male	58	Body/tail	Present	2	> 2	N0	Ⅰ B	-	-
15-10223	Male	60	Head	Absent	3	> 2	Nx	Ⅱ B	6	1
15-12250	Male	78	Head	Present	3	> 2	Nx	Ⅱ B	22	1
15-13833	Male	77	Body/tail	Present	2	> 2	Nx	Ⅱ B	-	-
15-14783	Male	78	Head	Present	2	⩽2	N0	Ⅰ A	50	0
15-15056	Male	76	Head	Present	2	⩽2	N0	Ⅰ A	5	1
15-18255	Male	75	Head	Present	2	> 2	N0	Ⅰ B	10	1
15-22355	Female	71	Body/tail	Present	3	> 2	N0	Ⅰ B	-	-
15-25006	Male	64	Body/tail	Present	2	> 2	Nx	Ⅱ B	37	1
15-25311	Male	51	Head	Present	3	> 2	Nx	Ⅱ B	24	1
15-41136	Male	53	Head	Present	3	> 2	Nx	Ⅱ B	36	1
15-37895	Female	66	Head	Present	2	> 2	N0	Ⅰ B	48	0
15-25726	Male	77	Head	Present	3	> 2	N0	Ⅰ B	-	-
15-11421	Male	64	Head	Present	2	> 2	N0	Ⅰ B	52	0
15-11998	Female	64	Body/tail	Absent	3	> 2	N0	Ⅱ A	12	1
